# Inhibition of XPO1 with KPT-330 induces autophagy-dependent apoptosis in gallbladder cancer by activating the p53/mTOR pathway

**DOI:** 10.1186/s12967-022-03635-w

**Published:** 2022-09-30

**Authors:** Cheng Zhao, Zi-yi Yang, Jian Zhang, Ou Li, Shi-lei Liu, Chen Cai, Yi-jun Shu, Li-jia Pan, Wei Gong, Ping Dong

**Affiliations:** 1 Laboratory of General Surgery and Department of General Surgery, Xinhua Hospital Affiliated With Shanghai Jiao Tong University School of Medicine, No. 1665 Kongjiang Road, Shanghai, 200092 China; 2 Shanghai Key Laboratory of Biliary Tract Disease Research, No. 1665 Kongjiang Road, Shanghai, 200092 China

**Keywords:** Chromosome region maintenance 1, Gallbladder cancer, KPT-330, Apoptosis, Autophagy

## Abstract

**Background:**

Gallbladder cancer (GBC) is a highly aggressive malignant cancer in the biliary system with poor prognosis. XPO1 (chromosome region maintenance 1 or CRM1) mediates the nuclear export of several proteins, mainly tumor suppressors. Thus, XPO1 functions as a pro-oncogenic factor. KPT-330 (Selinexor) is a United States Food and Drug Administration approved selective inhibitor of XPO1 that demonstrates good therapeutic effects in hematologic cancers. However, the function of XPO1 and the effect of KPT-330 have not been reported in GBC.

**Methods:**

We analyzed the correlation between XPO1 expression levels by q-PCR and clinical features of GBC patients. Cell proliferation assays were used to analyze the in vitro antitumor effects of XPO1 inhibitor KPT-330. mRNA sequencing was used to explore the underlying mechanisms. Western blot was performed to explore the relationship between apoptosis and autophagy. The in vivo antitumor effect of KPT-330 was investigated in a nude mouse model of gallbladder cancer.

**Results:**

We found that high expression of XPO1 was related to poor prognosis of GBC patients. We observed that XPO1 inhibitor KPT-330 inhibited the proliferation of GBC cells in vitro. Furthermore, XPO1 inhibitor KPT-330 induced apoptosis by reducing the mitochondrial membrane potential and triggering autophagy in NOZ and GBC-SD cells. Indeed, XPO1 inhibitor KPT-330 led to nuclear accumulation of p53 and activated the p53/mTOR pathway to regulate autophagy-dependent apoptosis. Importantly, KPT-330 suppressed tumor growth with no obvious toxic effects in vivo.

**Conclusion:**

XPO1 may be a promising prognostic indicator for GBC, and KPT-330 appears to be a potential drug for treating GBC effectively and safely.

**Supplementary Information:**

The online version contains supplementary material available at 10.1186/s12967-022-03635-w.

## Introduction

Gallbladder cancer (GBC) is the most common and aggressive malignant tumor of the human biliary system [1]. Due to the lack of typical clinical manifestations and early diagnostic tools, many patients are diagnosed at an advanced stage [2]. Up to now, the only treatment for GBC has been surgical removal, and even than only 10% of patients at the early stages have access to surgery [3, 4]. Thus, patients with advanced GBC have rapid tumor progression, and their prognosis is very poor, with a 5-year survival rate of only 5% [5]. Although many chemotherapies and targeted therapies are now available, the 5-year survival rate has not improved significantly [6]. Therefore, it is extremely urgent to discover more effective molecular targets and therapeutic modalities for the treatment of GBC.

Nucleocytoplasmic transport of macromolecules is an important means for tumor cells to survive and proliferate [7]. Chromosome region maintenance 1 (XPO1, exportin 1, or CRM1) belongs to the karyopherin beta family, and is the predominant receptor mediating nucleocytoplasmic transport [8, 9]. XPO1 is responsible for transporting most of the tumor suppressors and growth regulators; up to 220 proteins bearing the nuclear export signal (a leucine-rich region) are recognized by XPO1 [10]. XPO1 is overexpressed in a variety of tumors, including pancreatic cancer, osteosarcoma, glioma, cervical cancer, and hematological malignancies [8]. Therefore, inhibition of XPO1 has the potential to be an effective anticancer therapeutic approach [11, 12].

Leptomycin B specifically recognizes and blocks XPO1 and inhibits cells growth in different tumor cell lines; unfortunately, its strong toxicity limits its clinical application [13]. This inspired scientists to develop new selective inhibitors of nuclear export (SINEs). KPT-330 (Selinexor), a novel oral SINE, has been approved by the United States Food and Drug Administration for the treatment of refractory multiple myeloma and relapsed/refractory diffuse large B-cell lymphoma. Mechanistically, KPT-330 prevents the nucleocytoplasmic transport activity of XPO1, leading to accumulation of molecules that it transports in the nucleus [14–17]. However, the biological function of XPO1 in GBC, and the therapeutic effect of KPT-330 in GBC, have not yet been evaluated.

In this study, we investigated the biological function of XPO1 in GBC and the anti-cancer effects of the XPO1 inhibitor, KPT-330. Our study is the first to demonstrate the significant association between overexpression of XPO1, and poor clinical characteristics and prognosis in GBC patients. It was found that XPO1 inhibitor KPT-330 inhibited proliferation and colony formation of GBC cells. XPO1 inhibitor KPT-330 also induced mitochondria-dependent apoptosis and autophagy of GBC cells. Furthermore, KPT-330 activated the p53/mTOR pathway to induce autophagy-dependent apoptosis. In a nude mouse model of GBC, KPT-330 inhibited tumor growth without causing obvious toxic effects. Overall, XPO1 is a promising prognostic indicator for GBC, and KPT-330, a specific inhibitor of this transporter, might be a more effective and less cytotoxic chemotherapeutic agent for the treatment of GBC.

## Materials and methods

### Patients and clinicopathological data

Gallbladder cancer tissues and cholecystitis tissue were obtained from the Department of General Surgery in Xinhua Hospital affiliated with Shanghai Jiao Tong University School of Medicine (Shanghai, China). No patient underwent chemotherapy, radiotherapy, or immunotherapy before surgery. This study received the approval of the Ethics Committee of Xinhua Hospital, and all patients provided informed consent. All specimens were confirmed by pathological diagnosis. The cancer stage of each patient was assessed according to the 8th edition of the American Joint Committee on Cancer Staging Manual.

### Immunohistochemistry (IHC) and scoring

Immunohistochemistry (IHC) staining was conducted using the Servicebio Technology (Wuhan, China). Immunohistochemistry was conducted according to a standard protocol The IHC score was calculated according to the staining extent and intensity as follows: Negative (1): < 10% immunoreactive cells; Weak (2): 10%-49% immunoreactive cells; Moderate (3): 50%-74% immunoreactive cells; Strong (4): ≥ 75% immunoreactive cells. The staining color was scored as no staining (0), light yellow (1), brownish yellow (2) and brown (3). The final score was calculated by multiplying the two scores. Involved antibodies were given in Table 1.

**Table 1:** Information of antibody

Antibody	Catalogue	Dilution	Company
XPO1	46,249	1:2000	Cell Signaling Technology, USA
PCNA	13110S	1:2000	Cell Signaling Technology, USA
Ki67	9129S	1:2000	Cell Signaling Technology, USA

### Cell culture and reagents

The human GBC cell lines (NOZ and GBC-SD) were obtained from the cell bank of Shanghai Institutes for Biological Sciences, Chinese Academy of Sciences (Shanghai, China). Cells were cultured in DMEM medium (Gibco) and were supplemented 10% fetal bovine serum (Gibco), at 37 °C in a humidified chamber containing 5% CO2. KPT-330, rapamycin (treatment concentration of 0.4 μM and processing time of 6 h), chloroquine (treatment concentration of 0.2 μM and processing time of 6 h), Z-VAD-FMK (treatment concentration of 0.1 μM and processing time of 6 h), and MHY1485 (treatment concentration of 0.5 μM and processing time of 6 h) were obtained from Selleck Chemicals (Houston).

### TdT-mediated dUTP nick-end labeling assay

One Step TUNEL Apoptosis Assay Kit (Beyotime) was used to conduct TdT-mediated dUTP nick-end labeling (TUNEL) assays according to the manufacturer’s instructions. The cells were examined under a fluorescence microscope (Leica).

### Flow cytometry

Flow cytometry was used to conduct the apoptosis, cell cycle assays and mitochondrial membrane potential assay. NOZ and GBC-SD cells were treated with KPT-330 for 48 h in the respective IC50 values. Annexin V-FITC kit (BD Biosciences, CatLog: 556,547), Cell Cycle Analysis Kit (Beyotime) and mitochondrial membrane potential assay kit with JC-1(Beyotime) were used according to the manufacturer’s protocol. In the end, the samples were analyzed using flow cytometry (CytoFLEX, Beckman Coulter).

### Cell transfection

The siRNAs were synthesized by Genomeditech (PRC). The siRNAs used were listed in Additional file 1: Table S1. RFect reagent (Baidai) was used for transfection according to the manufacturer’s protocol. In brief, cells were seeded in 6-well plates at about 30% density. siRNA was diluted to 50 nM with 50 uL of Opti-MEM (Gibco) and 5 uL of RFect (Baidai) was diluted with 50 uL of Opti-MEM (Gibco). Then transfection reagent dilution and siRNA dilution were mixed and left for 20 min at room temperature. The mixture was then added to the cell culture dish containing 1 mL of Opti-MEM (Gibco) and replaced with fresh medium after 6 h. After 48 h, cells were collected for further experiments.

### Quantitative real-time PCR

Total RNAs were extracted from tissue samples, NOZ and GBC-SD cells by using Trizol reagent (Invitrogen). cDNA was generated using the PrimeScript RT reagent kit with gDNA Eraser (TaKaRa) according to the manufacturer’s instructions. The primers used for amplification were listed in Additional file 1: Table S2. Target gene expression level was detected using SYBR Green method and the StepOnePlus Real-time PCR System (Applied Biosystems).

### Western blot analysis

Firstly, proteins were isolated with RIPA Lysis buffer (Beyotime) and quantified using BCA assay (Beyotime). Next, proteins were separated by SDS-PAGE and transferred onto PVDF membranes (Millipore). Then, 5% skim milk was used to block the blots for 2 h at room temperature. A series of primary antibodies were added to the appropriate position of the PVDF membranes and incubated overnight at 4 °C. Finally, all blots reacted with the suitable HRP-conjugated secondary antibody (Beyotime) and the immunoreactive bands were detected by chemiluminescence and visualized using a Gel Doc 2000 (Bio-Rad). The list of antibodies is given in Table 2.

**Table 2:** Information of antibody

Antibody	Catalogue	Dilution	Company
GAPDH	5174	1:1000	Cell Signaling Technology, USA
XPO1	46,249	1:1000	Cell Signaling Technology, USA
p53	2527	1:1000	Cell Signaling Technology, USA
LC3-I/II	12,741	1:1000	Cell Signaling Technology, USA
Bcl-2	4223	1:1000	Cell Signaling Technology, USA
BAX	5023	1:1000	Cell Signaling Technology, USA
PARP	9532	1:1000	Cell Signaling Technology, USA
Cleaved-PARP	5625	1:1000	Cell Signaling Technology, USA
Caspase9	9504	1:1000	Cell Signaling Technology, USA
Cleaved-Caspase9	9509	1:1000	Cell Signaling Technology, USA
Caspase3	9662	1:1000	Cell Signaling Technology, USA
Cleaved-Caspase3	9664	1:1000	Cell Signaling Technology, USA
p-mTOR (Ser2448)	5536	1:1000	Cell Signaling Technology, USA
mTOR	2983	1:1000	Cell Signaling Technology, USA
p21	2947	1:1000	Cell Signaling Technology, USA
p27	3686	1:1000	Cell Signaling Technology, USA
H3	60,932	1:1000	Cell Signaling Technology, USA

### Ad-mCherry-GFP-LC3B transfection

Cells were seeded in 96-well plates and then transfected with Ad-mCherry-GFP-LC3B adenovirus (Beyotime) at an MOI of 4 for 6 h, following treatment with KPT-330 for 48 h in the respective IC50 values. Then, images were taken with a fluorescence microscope (Leica).

Viruses used in infection were calculated according to Eq.

$$\mathrm{Virus}\left(\mathrm{\mu L}\right)=\frac{\mathrm{MOI}\times \mathrm{Cell\, number}}{\mathrm{Virus\, titer}\left(\frac{\mathrm{PFU}}{\mathrm{mL}}\right)}\times 1000$$

### Cell proliferation assay and IC50 assay

The cell proliferation and IC50 assay were performed with a cell counting Kit-8 assay (CCK-8) (Yeasen). Approximately1000 of NOZ and GBC-SD cells were seeded in 96-well plates. The cell proliferation and IC50 value curves were plotted using absorbance at 450 nm. As for cell proliferation assay, after specific treatment, according to the manufacturer’s instructions, 10 μL CCK-8 solution and 100 μL culture medium were added to each well of the plate. The plate was incubated for 1.5 h in the incubator and the OD450 was measured by the microplate reader. As for IC50 assay, after cells were treated with KPT-330 for 48 h, 10 μL CCK-8 solution and 100 μL culture medium were added to each well of the plate. The plate was incubated for 1.5 h in the incubator and the OD450 was measured by the microplate reader.

### Colony formation assay

NOZ and GBC-SD cells were seeded in 6-well plates at a density of 1000 cells per well for 10 days. The cells were fixed with 4% paraformaldehyde and stained with 0.1% crystal violet. Only colonies with more than 50 cells were counted.

### mRNA Sequencing and bioinformatics analysis

mRNA sequencing and bioinformatics analysis of NOZ cells treated with KPT-330 for 48 h in IC50 value were conducted by BGI company (Shenzhen, China). Briefly, after filtering the sequencing data using SOAPnuke (v1.5.2, https://github.com/BGI-flexlab/SOAPnuke), clean reads were obtained in FAtSTQ format [18]. Firstly, to calculate the gene expression levels of each sample, we used Bowtie2 (v2.2.5) to align clean reads to the reference gene sequences and then used RSEM (v1.2.8), while differential gene expression was analyzed by using DESeq2(v1.4.5) under the condition of |${Log}_{2}$ FC|≥ 2, and Q-value ≤ 0.05 [19, 20]. Based on differential gene expression, pheatmap (v1.0.8) was used to draw a heatmap (https://cran.r-project.org/web/packages/pheatmap/). Then, to conduct GO analysis (http://www.geneontology.org/) and KEGG analysis (https://www.kegg.jp/), Phyper (https://stat.ethz.ch/R-manual/R-devel/library/stats/html/Hypergeometric.html) was used to calculated P value, which was then corrected for multiple testing by Q-value (https://bioconductor.org/packages/release/bioc/html/qvalue.html) [21].

### Xenograft model

Male nude mice were purchased from the Shanghai Laboratory Animal Center of the Chinese Academy of Sciences (Shanghai, China). The in vivo study was approved by the Ethics Committee of Xinhua Hospital. To establish a subcutaneous xenograft model, NOZ cells (2 × ${10}^{6}$ cells in 100 µL PBS) were injected subcutaneously in two groups of nude mice (6 mice/group). The two groups were treated via oral gavage once every two days: vehicle (0.3% CMC-Na) or KPT-330 (20 mg/kg) dissolved in vehicle (0.3% CMC-Na). Tumor volumes (1/2 × ${width}^{2}$  × length) were measured weekly using a caliper. After 4 weeks of treatment, tumors and tissues were collected for further assays.

### Statistical analysis

Statistical analyses were performed with Prism 8 (GraphPad Software) and SPSS 24 (SPSS Inc.). Student's t test was performed between two groups and analysis between multiple groups was conducted by one-way analysis of variance, data are recorded int the form of mean ± SD. P values of < 0.05 were considered statistically significant (p < 0.05: *, p < 0.01: **, p < 0.001: ***). We also use the Pearson’s χ2 test to detect the correlation between XPO1 expression and clinicopathologic data. Also, Kaplan–Meier test for the univariate survival analysis.

## Results

### Overexpression of XPO1 correlated to poor prognosis of GBC patients

**Fig. 1:**
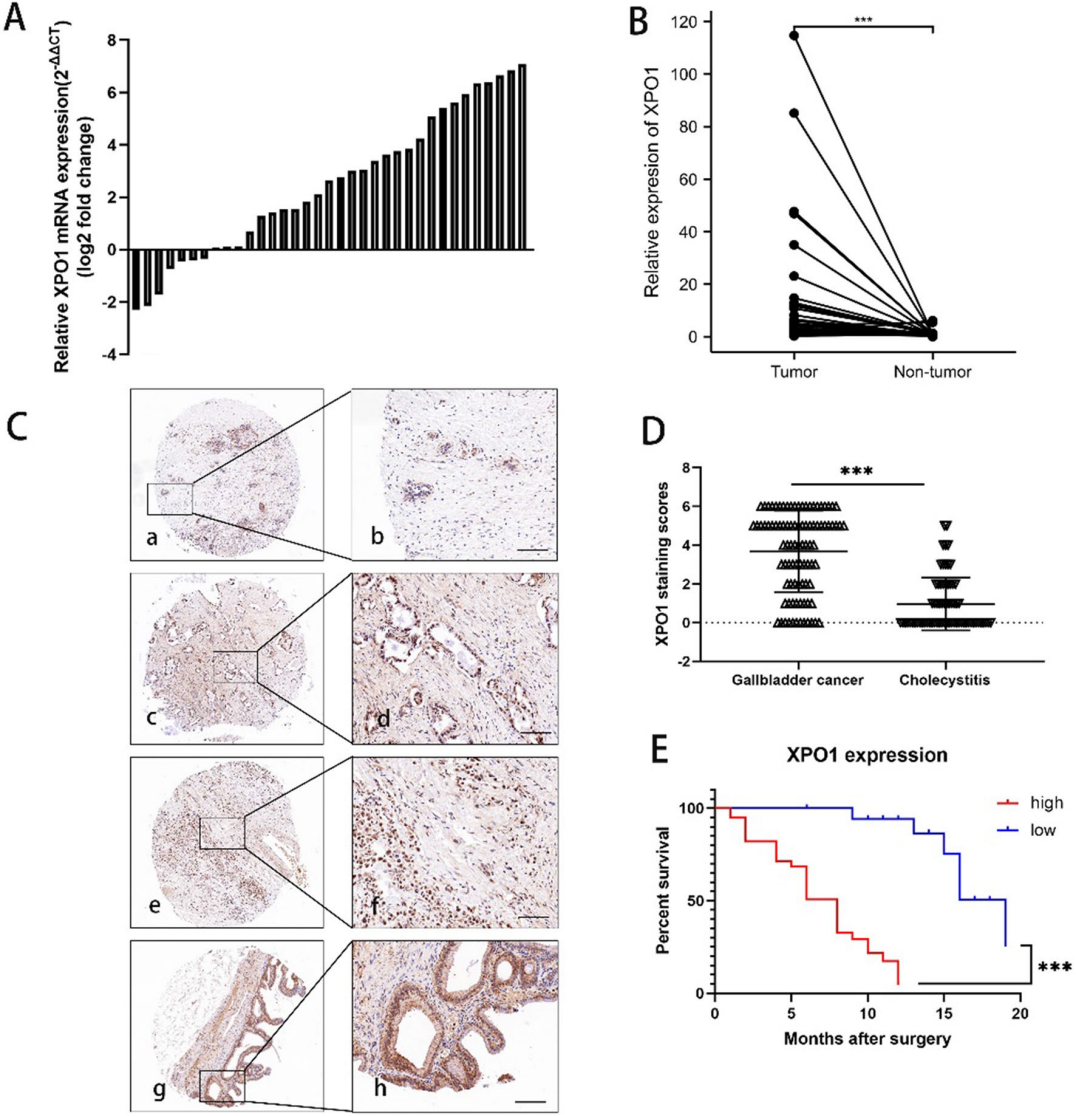
Overexpression of XPO1 was correlated to poor prognosis of GBC patients. **A** The comparison of XPO1 mRNA expression level between GBC tumor tissues and adjacent non-tumor tissues using qRT-PCR (n = 35). **B** Relative XPO1 expression level in GBC tissues and their corresponding non-tumor tissues (2 − ΔCT) (n = 35). Student's t test was applied to the statistical analysis. **C** Representative images of GBC IHC staining with an anti-XPO1 antibody. (a, b) Negative expression of XPO1 in cholecystitis tissues; (c, d) weak expression of XPO1 in GBC tissues; (e, f) moderate expression of XPO1 in GBC tissues; (g, h) strong expression of XPO1 in GBC tissues. Scale bars represent 100 μm. **D** Average staining scores for XPO1 expression in GBC tumor tissues (n = 92) and Cholecystitis tissues (n = 69) (P < 0.001) tested by Student's t test. **E** Patients with high XPO1 protein expression present shorter overall survival time assessed by Kaplan–Meier test

To clarify the expression of XPO1 in GBC, we first examined its mRNA level in 35 pairs of GBC and adjacent non-cancer tissues by the qRT-PCR. The results showed that XPO1 mRNA expression was significantly increased in GBC tissues compared with that in corresponding non-cancer tissues assessed by Student's t test (Fig. 1A and B). We also assessed XPO1 protein expression in 94 tissue samples of GBC and 69 cases of cholecystitis that were established as control by IHC (Fig. 1C). As shown in Fig. 1D, XPO1 was barely detectable in most of cholecystitis specimens compared with cancer samples calculated by Student's t test. To analyze the correlation between XPO1 expression levels and clinicopathological data of GBC patients, we used 57 samples from GBC patients with clinical follow-up data.

Based on the fold change, we divided patients into high level of XPO1 (fold change ≥ 2) and low level of XPO1 (fold change < 2). High level of XPO1 was closely associated with pathologic T stage (P < 0.001), lymph node metastasis (P = 0.047), and TNM stage (P = 0.001) evaluated by Pearson’s χ2 test (Table 3). Furthermore, the overall survival time of GBC patients after surgery with high XPO1 expression was significantly shorter than patients with low XPO1 expression assessed by Kaplan–Meier test (Fig. 1E).

**Table 3:** Association of XPO1 expression with the clinicopathological characteristics of GBC

Characteristic	Cases	XPO1 expression		P-value
		Low	High	
Sex				0.974
Male	16	5	11	
Female	41	13	28	
Age (years)				0.423
< 60	15	6	9	
≥ 60	42	12	30	
Histology				0.407
Moderate/WelI	45	13	32	
Poor	12	5	7	
T class				< 0.001
Tis/T1/T2	19	13	4	
T3/T4	38	5	35	
Lymph node metastasis				0.048
Present	21	13	11	
Absent	36	5	28	
Metastasis				0.081
Negative	51	18	33	
Positive	6	0	6	
TNM				0.001
I ~ II	13	9	4	
III ~ IV	44	9	15	

Pearson’s χ2 test was applied to detect the correlation between XPO1 expression and clinicopathologic data

### XPO1 inhibitor KPT-330 inhibited proliferation and induced G0/G1 cell cycle arrest of GBC cells

**Fig. 2:**
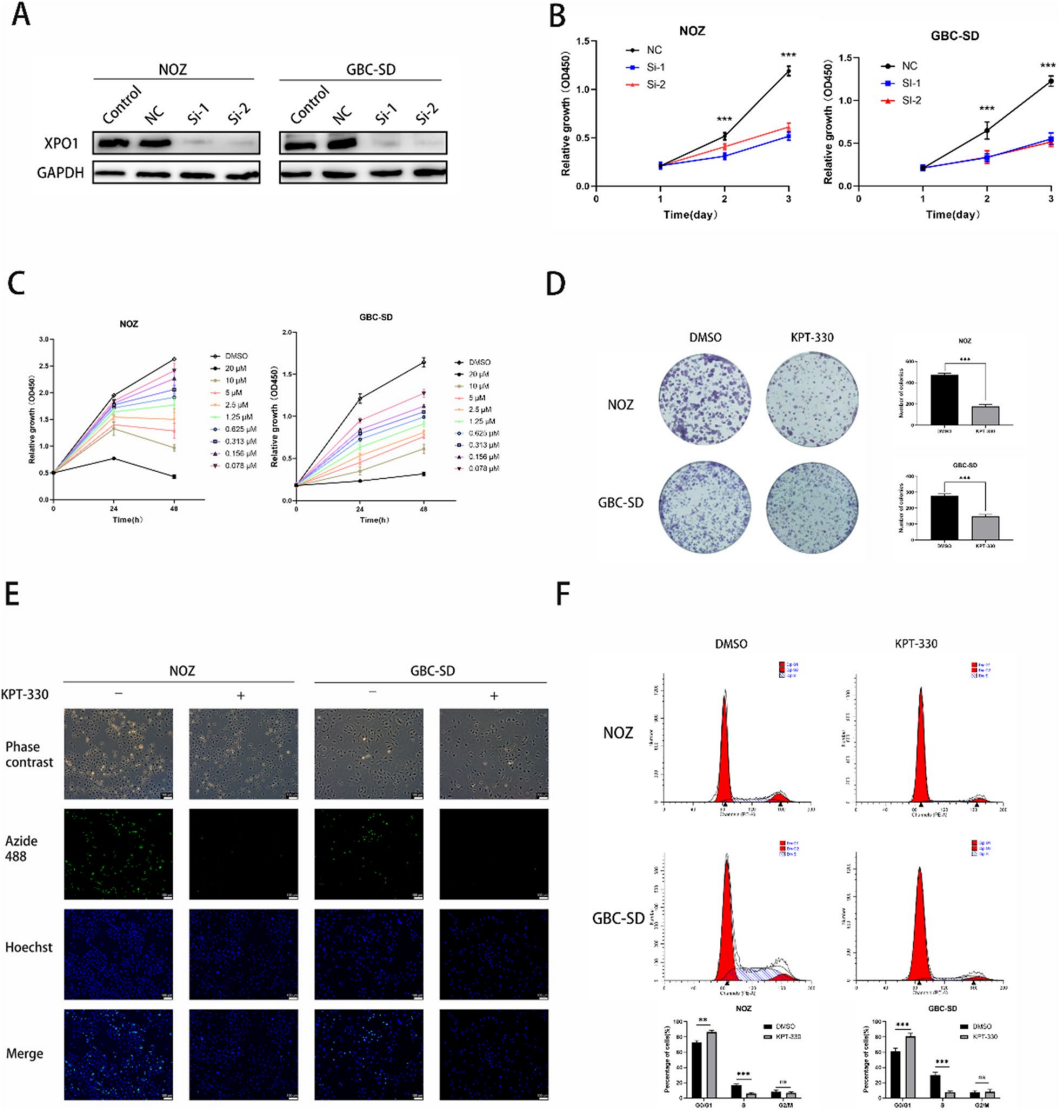
XPO1 inhibitor KPT-330 inhibited proliferation and induced G0/G1 cell cycle arrest of GBC cells. **A** The XPO1 expression after siRNAs transfected NOZ and GBC-SD was detected by western blot. “NC” means “Negative Control” group transfected by NC-siRNA. “Control” means “untransformed cells” group. **B** The proliferation of NOZ and GBC-SD cells was inhibited by XPO1 siRNAs transfection assessed by CCK-8 assay. **C** Proliferation of NOZ and GBC-SD detected by CCK-8 assay was inhibited in dose- and time-dependent manners by KPT-330. **D** Colony formation after KPT-330 treatment for 48 h in NOZ and GBC-SD was inhibited. **E** Fluorescence images of EdU-488 showed reduced green fluorescence after KPT-330 treatment relative to the DMSO treatment group. Scale bars represent 100 μm. **F** KPT-330 induced G0/G1 phase cell cycle arrest in NOZ and GBC-SD was detected by flow cytometry. Student's t test was applied to the statistical analysis in this figure. Data presented as mean ± SD (n = 3)

To determine the role of XPO1 in GBC cells, we knocked down XPO1 by siRNAs. And the two different XPO1 siRNAs significantly decreased XPO1 at the protein level (Fig. 2A). The results in Fig. 2B showed that the proliferation of NOZ and GBC-SD cells was significantly inhibited by XPO1-siRNAs evaluated by Student's t test. This finding tentatively suggested that inhibition of XPO1 could inhibit cell proliferation, then we treated GBC cells with KPT-330, which could specifically inhibit XPO1. The proliferation of NOZ and GBC-SD cells was significantly inhibited by XPO1 inhibitor KPT-330 both time and concentration dependently (Fig. 2C). The KPT-330 IC50 values for NOZ and GBC-SD cells at 48 h were 3.47 and 1.84 μM, respectively (Additional file 1: Fig. S1A); these concentrations and KPT-330 processing time (48 h) were used for subsequent experiments. The western blot results showed that KPT-330 significantly reduced the expression of XPO1 (Additional file 1: Fig. S1B). The colony formation assay showed that KPT-330 significantly reduced the number and size of colonies formed by GBC cells assessed by Student's t test (Fig. 2D). Similarly, the EdU-488 DNA synthesis assay showed the inhibitory effect of KPT-330 on the proliferation of NOZ and GBC-SD cells (Fig. 2E). Finally, we examined the effect of KPT-330 on the cell cycle in GBC cells by using flow cytometry. The results showed that KPT-330 increased the number of cells in the G0/G1 phase, with a reduced S phase population, compared with the DMSO group (P < 0.05) calculated by Student's t test (Fig. 2F). These results indicated that XPO1 inhibitor KPT-330 inhibited proliferation of GBC cells and caused G0/G1 cell cycle arrest.

### XPO1 inhibitor KPT-330 induced changes in multiple tumor pathways related to the transport function of XPO1

**Fig. 3:**
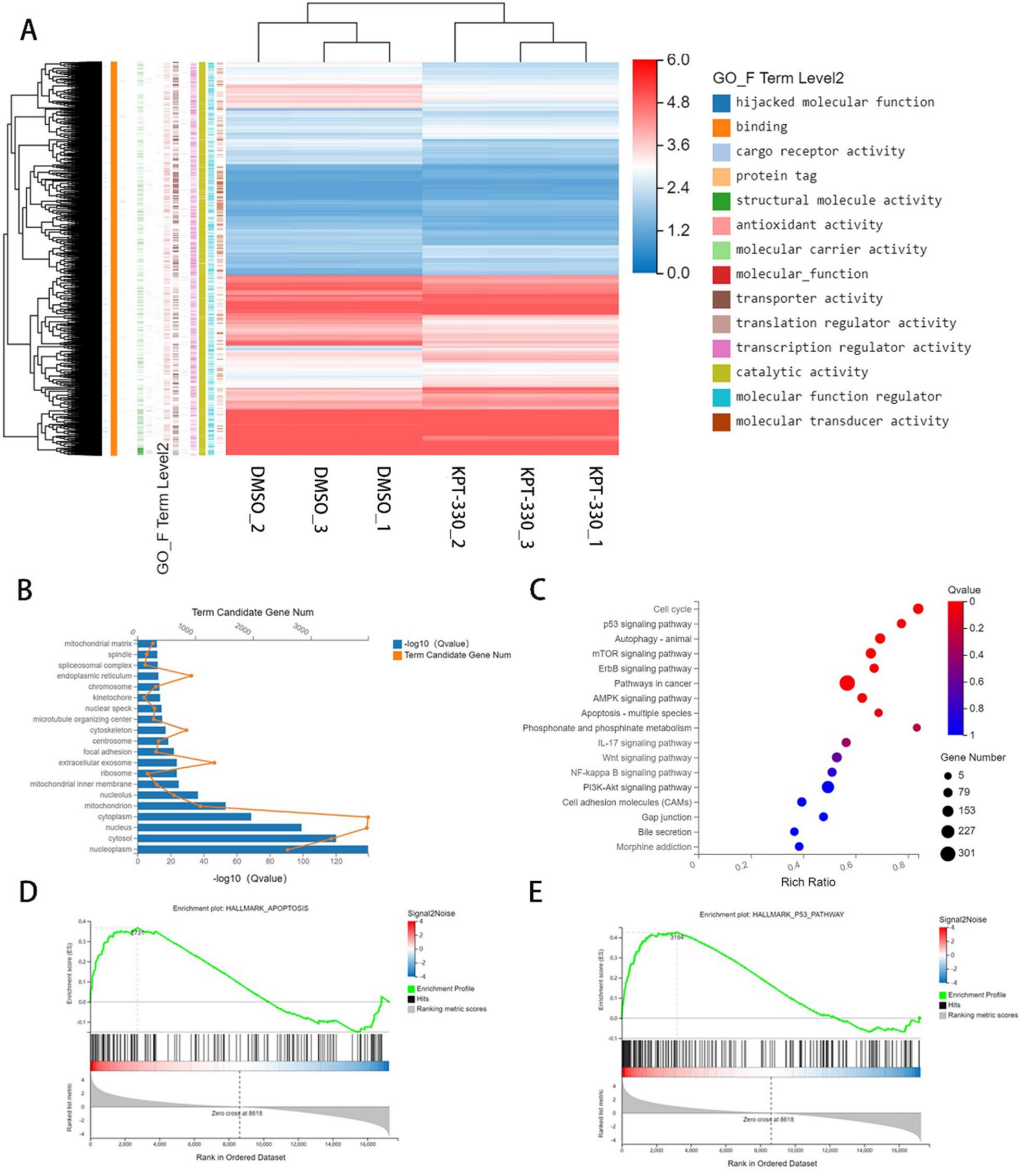
XPO1 inhibitor KPT-330 induced changes in multiple tumor pathways related to the transport function of XPO1. **A** Heatmap and Go function analysis of differential genes (|${Log}_{2}FC$|≥ 2 and Q-value ≤ 0.05) of NOZ after KPT-330 treatment for 48 h. **B** GO cellular component analysis of differential genes. **C** KEGG pathway analysis of differential genes. **D** Apoptosis pathway GSEA analysis of differential genes. E. p53 pathway GSEA analysis of differential genes

To clarify the role of XPO1 in GBC, we extracted total RNA from NOZ cells treated with KPT-330 for 48 h and performed an RNA-seq assay. We set |${log}_{2}FC$|≥ 2 and the Q-value ≤ 0.05 as inclusive criteria. The results showed that 838 genes were upregulated and 333 were downregulated (Additional file 1: Fig. S1C). According to the Gene Ontology (GO) analysis, the differentially expressed genes were closely associated with “cargo receptor activity”, “binding”, “molecular carrier activity”, and “transporter activity” (Fig. 3A). The GO cellular component showed that the differentially expressed genes were related to “nucleoplasm”, “cytosol”, “nucleus”, and “cytoplasm” (Fig. 3B). Thus, GO analysis results revealed changes were closely related to the role of XPO1 in nucleocytoplasmic transport. The KEGG results suggested important changes in “p53 signaling pathway”, “autophagy”, “mTOR signaling”, and “apoptosis” genes according to the Q-value (Fig. 3C). The GSEA also suggested significant changes in the p53 and apoptosis pathways (Fig. 3D and E). Overall, these bioinformatics analysis results indicated that XPO1 inhibitor KPT-330 could induce important changes related to cancer pathways in GBC cells.

### XPO1 inhibitor KPT-330 induced GBC cells apoptosis by reducing mitochondrial membrane potential

**Fig. 4:**
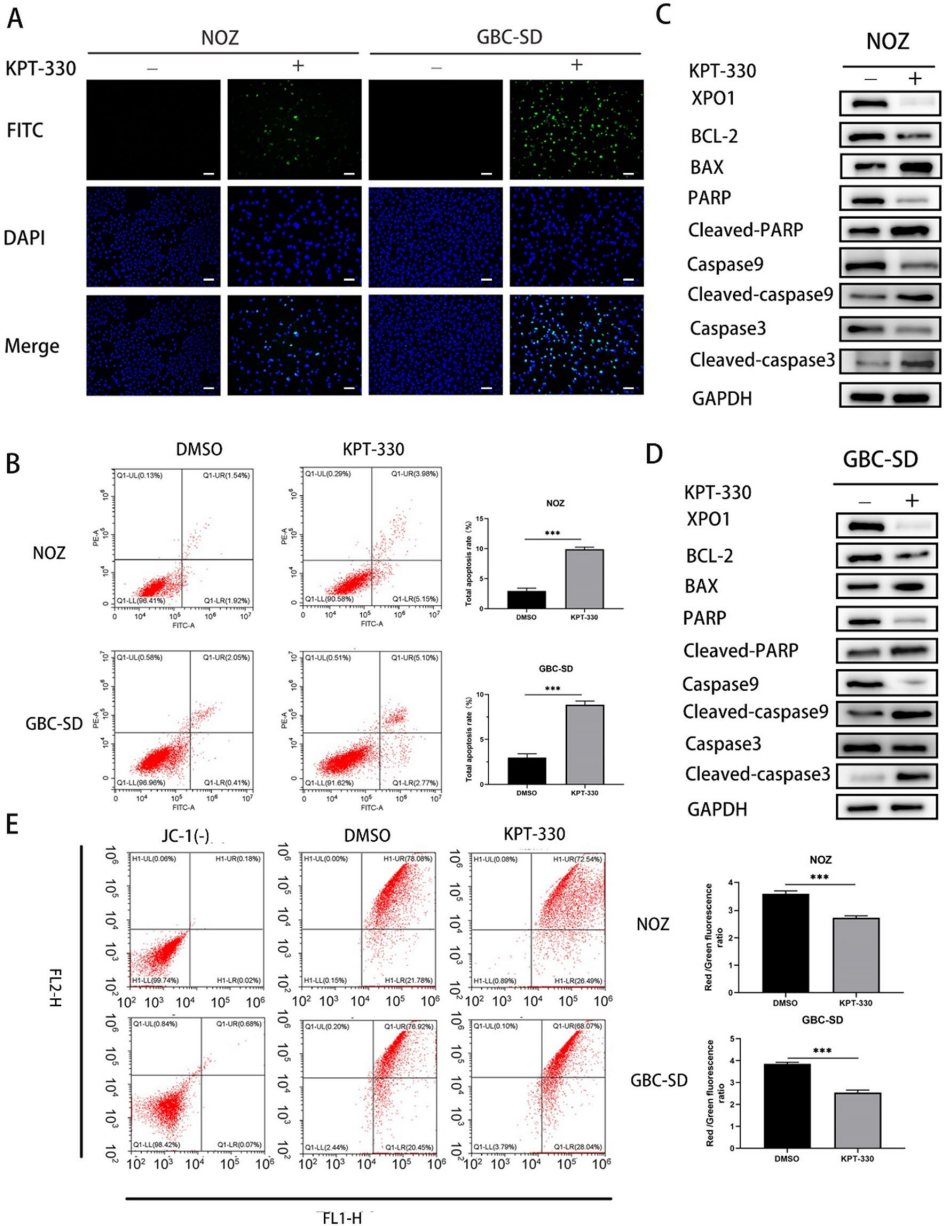
XPO1 inhibitor KPT-330 induced GBC cells apoptosis by reducing mitochondrial membrane potential. **A** Fluorescence images of TUNEL in NOZ and GBC-SD showed increased green fluorescence after KPT-330 treatment relative to the DMSO treatment group. Scale bars represent 100 μm. **B** Flow cytometry using PI/Annexin V-FITC double staining showed KPT-330 induced apoptosis of NOZ and GBC-SD. **C** and **D** Expression of BAX, Bcl-2, Cleaved PARP/PARP, Cleaved caspase 9/caspase 9, Cleaved caspase 3/caspase 3 was detected by western blot. **E** Flow cytometry analysis by using JC-1 staining. The horizontal axis channel “FL1-H” is the same as the FITC channel; the vertical axis channel “FL2-H” is the same as the PI channel. Student's t test was applied to the statistical analysis in this figure. Data presented as mean ± SD (n = 3)

In the bioinformatics analyses, we found significant changes in apoptosis-related pathways. To verify the existence of apoptosis, we utilized a variety of assays. First, the TUNEL assay confirmed that KPT-330 induced apoptosis in NOZ and GBC cells (Fig. 4A). In addition, the percentages of cells in both early and late apoptosis were significantly increased, as shown by flow cytometry, in NOZ and GBC-SD cells treated with KPT-330 and stained with PI and Annexin-V-FITC calculated by Student's t test (Fig. 4B). The expression of proteins associated with apoptosis was evaluated by western blot. The results indicated that the level of cleaved caspase-3 and caspase-9 proteins was increased in GBC cells treated with KPT-330, which activated cleaved PARP, an apoptosis marker (Fig. 4C and D). Further studies assessed the expression of apoptosis-related Bcl-2 family proteins. The results showed that the expression of Bax was remarkably increased, while the expression of Bcl-2 was significantly reduced (Fig. 4C and D).

A decrease in the ΔΨm, which reflects mitochondrial function, is a hallmark event of early apoptosis. According to the previously mentioned cell component of the GO analysis, which revealed changes in mitochondria and the mitochondrial inner membrane, we hypothesized that apoptosis in GBC cells treated with KPT-330 was associated with changes in ΔΨm. To address this hypothesis, NOZ and GBC-SD cells were treated with KPT-330 and stained with JC-1, which selectively enters mitochondria. The results showed that the ΔΨm was markedly diminished assessed by Student's t test (Fig. 4E). Together, these results indicated that XPO1 inhibitor KPT-330 induced GBC cells apoptosis through the mitochondria-mediated apoptosis pathway.

### XPO1 inhibitor KPT-330 induced autophagy of GBC cells through the mTOR pathway

**Fig. 5:**
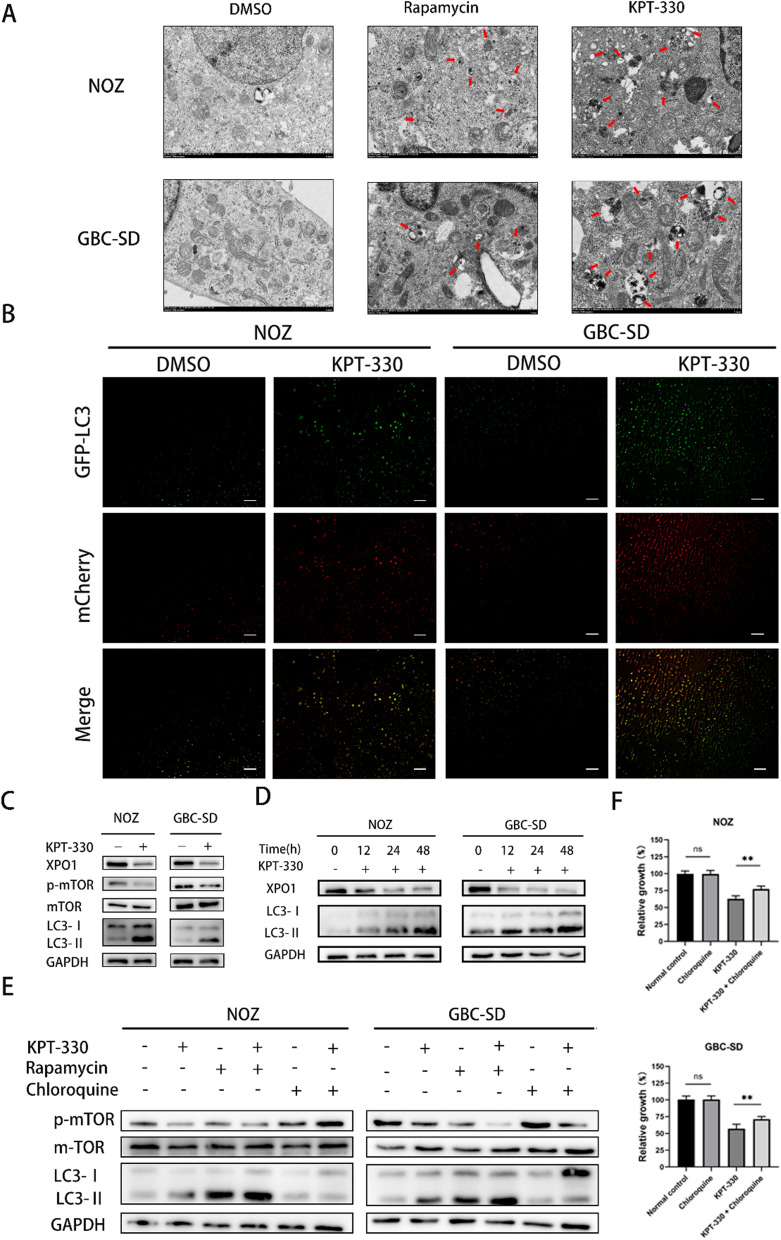
XPO1 inhibitor KPT-330 induced autophagy of GBC cells through the mTOR pathway. **A** Transmission electron microscopy in NOZ and GBC-SD showed more autophagosomes and autolysosomes after KPT-330 treatment compared with DMSO treatment group. Red arrows point to autophagosomes and autolysosomes. Scale bars represent 1.0 μm. **B** mCherry-GFP-LC3 dual fluorescent images indicated that green, red, and yellow (from the merged images) puncta increased greatly in KPT-330 treated cells compared to DMSO treatment in NOZ and GBC-SD. Scale bars represent 100 μm. **C** Expression of LC3- II/I and p-mTOR/mTOR was detected by western blot after KPT-330 treatment for 48 h. **D** Expression of LC3- II/I was detected by western blot after KPT-330 treatment at 0, 12, 24, 48 h. **E** Expression of LC3- II/I and p-mTOR/mTOR was detected by western blot after KPT-330 or rapamycin or chloroquine treatment. GBC cells were pre-treated with chloroquine (concentration of 0.2 μM) or rapamycin (concentration of 0.4 μM) for 6 h, then were treated with or without KPT-330 for 48 h. **F** Chloroquine attenuated inhibition effects of KPT-330 in NOZ and GBC-SD assessed by cell proliferation assays. GBC cells were pre-treated with chloroquine (concentration of 0.2 μM) for 6 h, then were treated with or without KPT-330 for 48 h. Student's t test was applied to the statistical analysis in this figure. Data presented as mean ± SD (n = 3)

The KEGG pathway analysis indicated that autophagy may exist in NOZ cells treated with KPT-330 (Fig. 3C). To determine whether KPT-330 induced autophagy, GBC cells were treated with KPT-330 for 48 h and their morphology was observed by transmission electron microscopy. GBC cells treated with rapamycin (which activated autophagy) with concentration of 0.4 μM for the same time period were used as a positive control. The results demonstrated KPT-330 and rapamycin treatment could be seen to form a similar autophagosomes and autolysosomes (red arrows point to) compared to control group (Fig. 5A). To assess the extent of autophagosome and autolysosome accumulation separately, we infected NOZ and GBC-SD cells with Ad-mCherry-GFP-LC3B. Green LC3 puncta represent mainly autophagosomes, while red LC3 puncta indicate both autophagosomes and autolysosomes, as mCherry maintains its fluorescence even in the acidic environment of lysosomes, while GFP loses its fluorescence [22]. The numbers of green, red, and yellow (from the merged images) puncta increased greatly in KPT-330 treated cells compared to control, implying an increase in autophagosomes and autolysosomes (Fig. 5B).

The transition from LC3-I to LC3-II is often considered a marker of autophagy, and can be used to monitor autophagosome formation at an early stage [23]. The LC3-II/LC3-I ratio increased with time in KPT-330 treated NOZ and GBC-SD cells (Fig. 5C and D). Meanwhile, we found that the p-mTOR/mTOR ratio was reduced. To further explore whether inhibition of XPO1 induced mTOR-mediated autophagy, we pre-treated GBC cells with the autophagy inhibitor, chloroquine (concentration of 0.2 μM), or the mTOR inhibitor, rapamycin (concentration of 0.4 μM) for 6 h, then treated cells with or without KPT-330 for 48 h. The results showed that the p-mTOR/mTOR ratio was reduced after treatment with either KPT-330 or rapamycin, and this reduction was enhanced by combining KPT-330 and rapamycin. Furthermore, chloroquine partially abrogated the reduction in the p-mTOR/mTOR ratio by KPT-330 (Fig. 5E). In addition, the cell proliferation results showed that chloroquine significantly prevented the decrease in proliferation caused by KPT-330 analyzed by Student's t test (Fig. 5F). Overall, these results suggested that the XPO1 inhibitor KPT-330 induced autophagy by regulating the mTOR pathway in GBC cells.

### XPO1 inhibitor KPT-330 activated p53/mTOR pathway to induce autophagy-dependent apoptosis

**Fig. 6:**
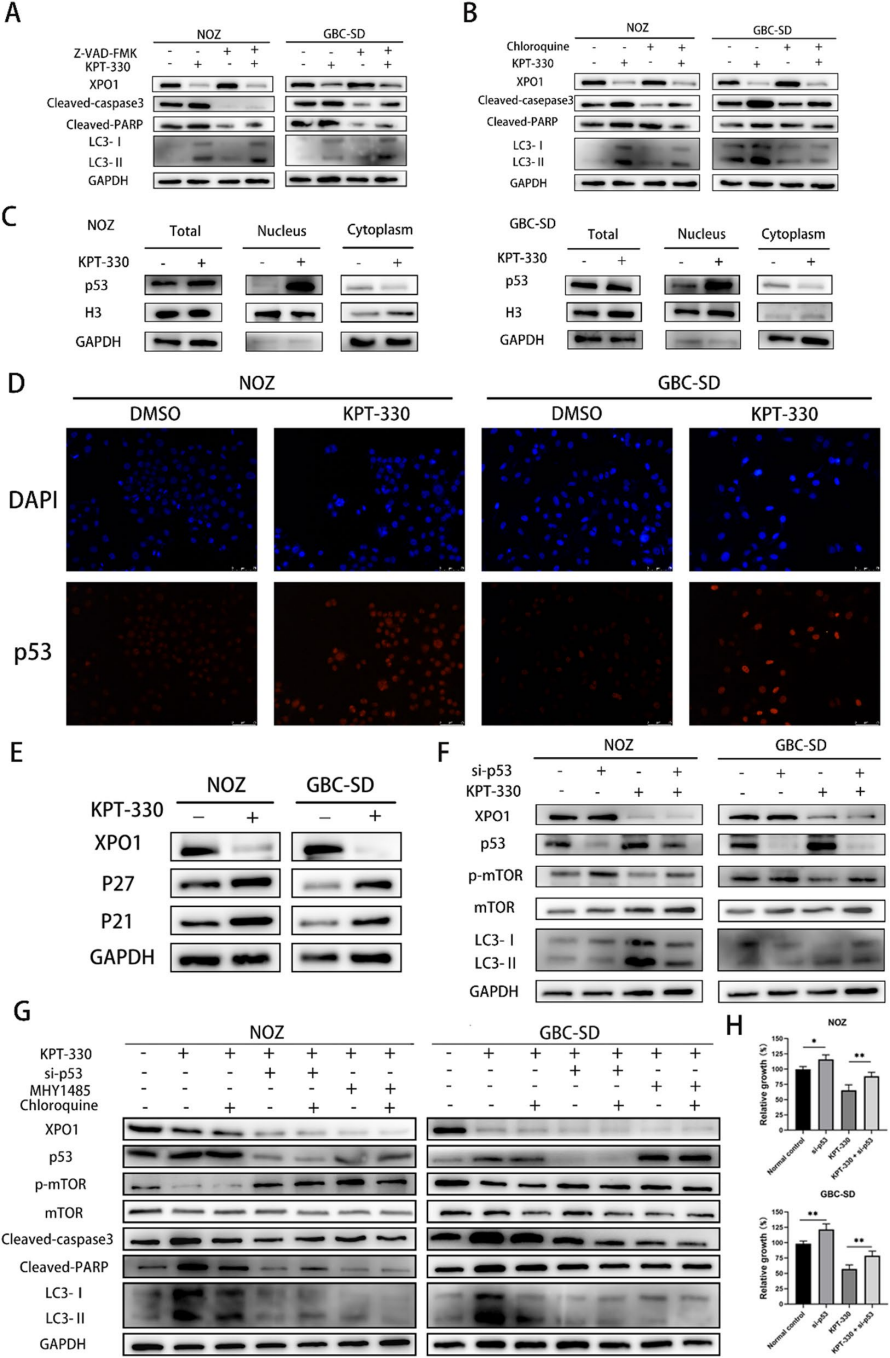
XPO1 inhibitor KPT-330 activated p53/mTOR pathway to induce autophagy-dependent apoptosis. **A** Expression of LC3- II/I, Cleaved PARP, Cleaved caspase 3 was detected by western blot after KPT-330 or Z-VAD-FMK treatment. Cells were pre-treated with Z-VAD-FMK (concentration of 0.1 μM) for 6 h, then were treated with KPT-330 for 48 h. **B** Expression of LC3- II/I, Cleaved PARP, Cleaved caspase 3 was detected by western blot after KPT-330 or chloroquine treatment. Cells were pre-treated with chloroquine (concentration of 0.2 μM) for 6 h, then were treated with KPT-330 for 48 h. **C** Western blot analysis of p53 levels in nucleus and cytoplasm of NOZ and GBC-SD showed that more p53 proteins were accumulated in nucleus after KPT-330 treatment. **D** Immunofluorescence images of p53 showed that more p53 proteins were accumulated in nucleus in NOZ and GBC-SD after KPT-330 treatment. Scale bars represent 75 μm. **E** P21 and P27 expression levels were detected by western blot after KPT-330 treatment for 48 h. **F** Expression of LC3- II/I, p53, p-mTOR/mTOR was detected by western blot after KPT-330 or p53-siRNA treatment. Cells were pre-treated with p53-siRNA for 48 h, then were treated with KPT-330 for 48 h. **G** Expression of LC3- II/I, p53, p-mTOR/mTOR, Cleaved PARP and Cleaved caspase 3 was detected by western blot. GBC cells were pre-treated with p53-siRNA for 48 h or MHY1485 (concentration of 0.5 μM) or chloroquine (concentration of 0.2 μM) for 6 h, then were treated with or without KPT-330 for 48 h. **H** Knockdown of p53 attenuated inhibition effects of KPT-330 in NOZ and GBC-SD assessed by cell proliferation assays. GBC cells were pre-treated with p53-siRNA for 48 h, then were treated with or without KPT-330 for 48 h. Student's t test was applied to the statistical analysis in this figure. Data presented as mean ± SD (*n* = 3)

It has been suggested that increased tumor cell apoptosis may be dependent on the presence of autophagy [24, 25]. To determine whether KPT-330 triggered autophagy decreased or increased apoptosis, the extent of apoptosis in NOZ and GBC-SD cells after chloroquine and/or KPT-330 treatment was examined by flow cytometry. The results indicated that the inhibition of autophagy by chloroquine partially abrogated KPT-330-induced apoptosis evaluated by Student's t test (Additional file 1: Fig. S1D). This result tentatively suggested that inhibition of autophagy may inhibit apoptosis induced by KPT-330. Then we conducted western blot for further analysis. Treatment of these cells with the cell-permeable broad-spectrum caspase inhibitor, Z-VAD-FMK (concentration of 0.1 μM), significantly decreased the levels of cleaved caspase-3 and PARP, whereas the increased LC3-II/LC3-I ratio caused by KPT-330 was not changed (Fig. 6A). Indeed, the rising cleaved forms of caspase-3 and PARP protein expression were effectively prevented by chloroquine in KPT-330 treated cells (Fig. 6B). In addition, the cell proliferation results showed that Z-VAD-FMK significantly prevented the decrease in proliferation caused by KPT-330 calculated by Student's t test (Additional file 1: Fig. S1E).

The KEGG pathway analysis and GSEA indicated significant changes in the p53 pathway. The western blot analysis of fractionated cell lysates showed that, p53 level was significantly increased in the nucleus but decreased in the cytoplasm after KPT-330 treatment of NOZ and GBC-SD cells (Fig. 6C). Moreover, immunofluorescence staining of p53 indicated that KPT-330 increased the nuclear accumulation of p53 in both NOZ and GBC-SD cells (Fig. 6D). p53 is involved in regulating cycle-related proteins including P21 and P27 [26]. The nuclear accumulation of p53 could activate its functions [27]. The western blot results confirmed that the expression of P21 and P27 was increased after KPT-330 treatment, which was consistent with the observed G0/G1 cell cycle arrest (Fig. 6E). It has been reported that p53 could affect the mTOR pathway and thereby affect cell autophagy [28–30]. We knocked down p53 by using siRNA (Additonal file 1: Fig. S1F). We treated cells with siRNAs for 48 h, then treated cells with KPT-330 for 48 h. This knockdown partially reduced the KPT-330-mediated increase in the phosphorylation level of mTOR, and the LC3-II/LC3-I ratio (Fig. 6F). These results indicated that KPT-330 induced nuclear accumulation of the p53 protein, which then activated the p53/mTOR pathway to regulate cell autophagy. To further confirm that KPT-330 regulation of the p53/mTOR pathway affects autophagy-dependent apoptosis, we performed TUNEL assays and western blot assays. The TUNEL assays indicated that inhibition of autophagy inhibited apoptosis induced by KPT-330 treatment, and inhibition of p53 by p53-siRNA or activation of mTOR by MHY1485 also inhibited apoptosis induced by KPT-330 treatment (Additional file 1: Fig. S2A). The results of western blot showed that inhibition of autophagy could decrease the rising level of cleaved caspase-3 and cleaved PARP protein in KPT-330 treated cells (Fig. 6G). Moreover, the changes of the phosphorylation level of mTOR and LC3-II/LC3-I ratio obtained after knockdown of p53 or activation of mTOR were consistent with the previous results. As shown in Fig. 6H, knockdown of p53 antagonized the anticancer effect of KPT-330 in terms of GBC cell growth assessed by Student's t test. In summary, XPO1 inhibitor KPT-330 induced the nuclear accumulation of p53 protein and activated p53/mTOR to induce autophagy-dependent apoptosis.

### *XPO1 inhibitor KPT-330 inhibited growth of GBC *in vivo* with excellent drug safety*

**Fig. 7:**
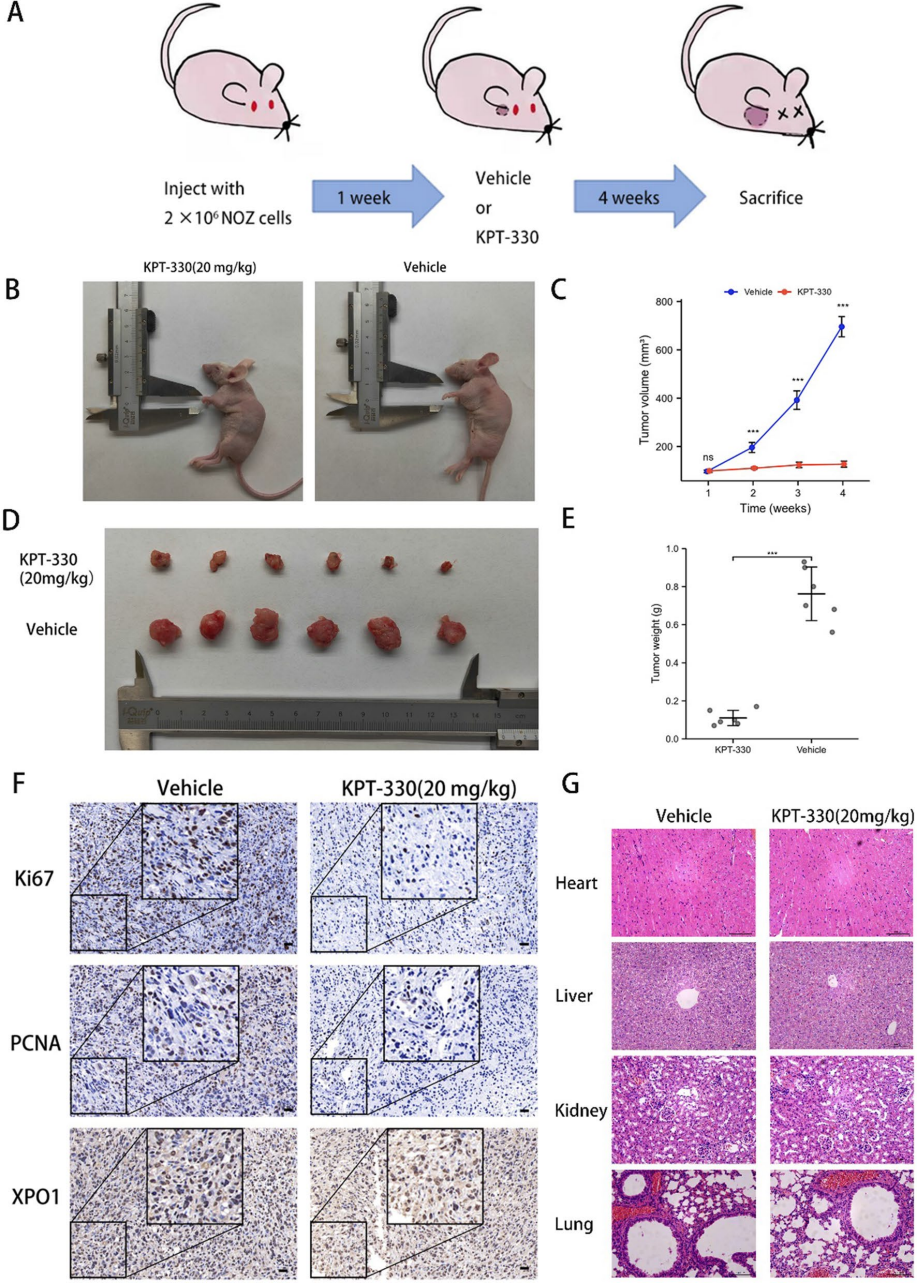
XPO1 inhibitor KPT-330 inhibited growth of GBC in vivo with excellent drug safety. **A** Schematic diagram of GBC xenograft mouse model treated with KPT-330 in vivo. **B** Photograph of sacrificed nude mice in KPT-330 treatment group and vehicle treatment group. **C** Removed subcutaneous tumors from KPT-330 treatment group and vehicle treatment group. **D** Tumor volume in different weeks in KPT-330 treatment group and vehicle treatment group. **E** Removed subcutaneous tumors weight in vehicle or KPT-330 group. **F** Immunohistochemistry showed that XPO1, Ki67 and PCNA expression levels of xenograft tumor tissues were lower in KPT-330 treatment group. Scale bars represent 200 μm. **G** No obvious pathological changes in the lung, liver, kidney and heart were found after KPT-330 treatment. Scale bars represent 100 μm. Student's t test was applied to the statistical analysis in this figure

To validate the anticancer effect of KPT-330 in vivo, we injected NOZ cells subcutaneously into the left flank of nude mice to construct a GBC tumor model. One week after injection, the mice were treated with 20 mg/kg KPT-330 for 4 weeks via oral gavage three times per week (Fig. 7A). Compared with vehicle-treated mice, the tumor volume and weight were significantly reduced following KPT-330 treatment calculated by Student's t test (Fig. 7B-E). IHC staining of tumor tissue showed that expression of the XPO1 protein was dramatically reduced in the KPT-330 treatment group. Similar results were obtained for IHC staining of Ki-67 and PCNA, whose expression is related to the proliferation of GBC cells evaluated by Student's t test (Fig. 7F and Additional file 1: Fig. S2B). In terms of safety, no changes were observed in the appearance and morphology of main organs (heart, liver, kidney and lung) of mice treated with KPT-330 (Fig. 7G). In addition, no obvious systemic toxicity was observed in routine blood examinations after KPT-330 treatment (Additional file 1: Table S3). Taken together, these results suggested that the XPO1 inhibitor, KPT-330, inhibited tumor growth in vivo with a good safety profile.

## Discussion

XPO1 is a key nuclear export carrier for a variety of tumor suppressor proteins [9]. Maintaining the balance of protein transport between the nucleoplasm is essential for controlling cell survival and death [31]. Thus, altering the nucleoplasmic localization of cargo proteins creates the opportunity to target a variety of unique pathways implicated in carcinogenesis [32]. For example, inhibiting XPO1 diminishes tumor growth and improves the efficacy of cisplatin in ovarian carcinoma [33, 34]. Inhibition of XPO1 also allows BCR-ABL to be trapped in the nucleus and sensitizes leukemic cells to the BCR-ABL inhibitor, imatinib, resulting in a strong decrease in their proliferative potential [35].

An important feature of tumors is the dysregulation of apoptosis. Consequently, apoptosis can be induced with various cytotoxic anticancer agents, making this one of the most effective strategies for the treatment of cancer [36]. Our previous studies and those of other researchers indicated that XPO1 inhibitors exerted its anticancer activities by inducing apoptosis in cancer cells [37–39]. Furthermore, XPO1 inhibitors can trigger p53-mediated caspase-dependent apoptosis in adult T-cell Leukemia [40]. In this study, we found that XPO1 inhibitor KPT-330 induced the nuclear accumulation of p53 protein.

In different cancer cells, autophagy can act as either a tumor suppressor or a tumor promoter, with the specific state depending on the cancer cell environment [41]. Our results showed that autophagy could be detected after KPT-330 treatment. Inhibition of the mTOR pathway has been reported to activate autophagy [42, 43]. Our bioinformatic and western blot analyses verified that KPT-330 could induce mTOR-mediated autophagy. Autophagy is part of a complex network that brings together oversight of metabolism, cell proliferation and apoptosis [44]. Several studies have confirmed that inducing autophagy can promote apoptosis in cancers [45, 46]. Our results confirmed that KPT-330 can induce autophagy-dependent apoptosis. Combined with the fact that tumor cells can undergo apoptosis upon KPT-330 treatment, and that KPT-330 leads to the development of autophagy-dependent apoptosis, we speculated that p53 could regulate autophagy. Based on this conjecture, we knocked down p53 by siRNA and showed that this knockdown reduced the extent of mTOR-mediated autophagy induced by KPT-330. We integrated KPT-330, p53-siRNA, MHY1485 and choroquine for furher experiments and the results confirmed that XPO1 inhibitor KPT-330 activated the p53/mTOR pathway to regulate autophagy-dependent apoptosis.

The in vivo experiment showed that the XPO1 inhibitor, KPT-330, had potent anti-GBC activity. Administration of 20 mg/kg of KPT-330 significantly inhibited the growth of GBC cells in a nude mouse xenograft model without any significant toxic effects. However, some clinical studies with KPT-330 have revealed adverse effects such as decreased appetite, vomiting, and decreased weight, but without significant effects on vital organs [47]. Further studies are needed to investigate in vivo effects of KPT-330 treatment.

In summary, our findings show that overexpression of XPO1 is significantly associated with poor prognosis in patients with GBC. Furthermore, XPO1 inhibitor KPT-330 can activate the p53/mTOR pathway to induce autophagy-dependent apoptosis in GBC cells. Therefore, XPO1 is a potential biomarker for the diagnosis of GBC, and KPT-330 might be a more effective and less cytotoxic chemotherapeutic agent for the treatment of GBC in the future.

## Supplementary Information

**Additional file 1: Table S1**. siRNA of genes. **Table S2**. Primer of genes. **Table S3**. Blood routine examination. **Figure S1**. A. Cell sensitivity of NOZ and GBC-SD cells was evaluated by CCK-8 assays. Cells were exposed to KPT-330 for 48h and KPT-330 IC50 values for NOZ and GBC-SD cells were 3.47 and 1.84 μM, respectively. B. The XPO1 expression was detected by western blot after KPT-330 for 48h in NOZ and GBC-SD. C. Volcano plots of differentially expressed genes under the control of |${log}_{2}FC$| ≥ 2 and the Q-value ≤ 0.05 after KPT-330 treatment in NOZ cells. D. Flow cytometry using PI/Annexin V-FITC double stain of NOZ and GBC-SD after KPT-330 or chloroquine treatment. Cells were treated with chloroquine (concentration of 0.2 μM) for 6h, then were treated with KPT-330 for 48h. E. Z-VAD-FMK attenuated inhibition effects of KPT-330 in NOZ and GBC-SD assessed by cell proliferation assays. Cells were treated with Z-VAD-FMK (concentration of 0.1 μM) for 6h, then were treated with KPT-330 for 48h. F. The p53 expression after siRNA transfected NOZ and GBC-SD was detected by western blot. “NC” means “Negative Control” group transfected by NC-siRNA. “Control” means “untransformed cells” group. Student's t test was applied to the statistical analysis in this figure. Data presented as mean ± SD (n = 3). **Figure S2**. A. Fluorescence images of TUNEL after KPT-330 or p53-siRNA or MHY1485 or chloroquine treatment in NOZ and GBC-SD. NOZ and GBC-SD cells were pre-treated with p53-siRNA for 48h or MHY1485 (concentration of 0.5 μM) or chloroquine (concentration of 0.2 μM) for 6h, then were treated with or without KPT-330 for 48h. Scale bars represent 100 μm. B. Immunohistochemistry score of XPO1, Ki67 and PCNA expression levels of xenograft tumor tissues in KPT-330 treatment or vehicle group. Student's t test was applied to the statistical analysis

## Data Availability

The mRNA-seq raw data can be found in SRA database: https://www.ncbi.nlm.nih.gov/bioproject/PRJNA827962. All data generated or analyzed during this study are included in this published article and its Additional files.

## Abbreviations

CCK8: Cell Counting Kit 8
GBC: Gallbladder cancer
GSEA: Gene Set Enrichment Analysis
IHC: Immunohistochemistry
KEGG: Kyoto Encyclopedia of Genes and Genomes
mTORL: mammalian target of rapamycin
PARP: Poly(ADP-ribose) polymerase
PCNA: Proliferating cell nuclear antigen
PI: Propidium iodide
qRT-PCR: Quantitative reverse transcription-polymerase chain reaction
SINEs: Selective inhibitors of nuclear export
TUNEL: Terminal deoxynucleotidyl transferase dUTP nick-end labeling; ΔΨm: mitochondrial membrane potential
XPO1: Chromosome region maintenance 1
NC: Negative control
TNM: Tumor Node Metastasis
T stage: Tumor stage
