# Supplementary material for: Inhibition of XPO1 with KPT-330 induces autophagy-dependent apoptosis in gallbladder cancer by activating the p53/mTOR pathway
Source: J Transl Med. 2022 Sep 30;20:434. doi: 10.1186/s12967-022-03635-w (PMC9524043; doi:10.1186/s12967-022-03635-w)
Supplement: Supplementary file 1 — Additional file 1: Table S1. siRNA of genes. Table S2. Primer of genes. Table S3. Blood routine examination. Figure S1. A. Cell sensitivity of NOZ and GBC-SD cells was evaluated by CCK-8 assays. Cells were exposed to KPT-330 for 48h and KPT-330 IC50 values for NOZ and GBC-SD cells were 3.47 and 1.84 μM, respectively. B. The XPO1 expression was detected by western blot after KPT-330 for 48h in NOZ and GBC-SD. C. Volcano plots of differentially expressed genes under the control of |${log}_{2}FC$log2FC| ≥ 2 and the Q-value ≤ 0.05 after KPT-330 treatment in NOZ cells. D. Flow cytometry using PI/Annexin V-FITC double stain of NOZ and GBC-SD after KPT-330 or chloroquine treatment. Cells were treated with chloroquine (concentration of 0.2 μM) for 6h, then were treated with KPT-330 for 48h. E. Z-VAD-FMK attenuated inhibition effects of KPT-330 in NOZ and GBC-SD assessed by cell proliferation assays. Cells were treated with Z-VAD-FMK (concentration of 0.1 μM) for 6h, then were treated with KPT-330 for 48h. F. The p53 expression after siRNA transfected NOZ and GBC-SD was detected by western blot. “NC” means “Negative Control” group transfected by NC-siRNA. “Control” means “untransformed cells” group. Student's t test was applied to the statistical analysis in this figure. Data presented as mean ± SD (n = 3). Figure S2. A. Fluorescence images of TUNEL after KPT-330 or p53-siRNA or MHY1485 or chloroquine treatment in NOZ and GBC-SD. NOZ and GBC-SD cells were pre-treated with p53-siRNA for 48h or MHY1485 (concentration of 0.5 μM) or chloroquine (concentration of 0.2 μM) for 6h, then were treated with or without KPT-330 for 48h. Scale bars represent 100 μm. B. Immunohistochemistry score of XPO1, Ki67 and PCNA expression levels of xenograft [file 12967_2022_3635_MOESM1_ESM.docx]

Supplementary materials

| Supplementary Table 1. siRNA of genes. | |
| --- | --- |
| Gene | siRNA |
| XPO1-siRNA1 | 5-AUUCGACUUGCGUACUCAAAUTT-3 |
| XPO1-siRNA2 | 5-CCUGCUUUCAAGGAACAUUUATT-3 |
| p53-siRNA | 5-CGGCGCACAGAGGAAGAGAAUTT-3 |
| NC-siRNA | 5- TTCTCCGAACGTGTCACGT-3 |

| Supplementary Table 2. Primer of genes. | | |
| --- | --- | --- |
| Gene | Forward primer | Reverse primer |
| XPO1 | 5-ATCTGACCCAACTTGTGTAGAGA-3 | 5-TGGTCCTACTTGCTCCAACAAT-3 |
| GAPDH | 5-CAACAGCCTCAAGATCATCAGC-3 | 5-TTCTAGACGGCAGGTCAGGTC-3 |

Supplementary Table3. Blood routine examination

| Indicator | Vehicle | KPT-330  (20mg/kg) | P-value |
| --- | --- | --- | --- |
| WBC | 5.917±1.204 | 6.000±0.969 | 0.8976 |
| Lymph | 3.583±0.825 | 3.617±1.053 | 0.9526 |
| Mon | 0.165±0.043 | 0.185±0.053 | 0.4930 |
| Gran | 1.250±0.532 | 0.983±0.355 | 0.3310 |
| Lymph% | 72.20±7.388 | 71.22±8.004 | 0.8294 |
| Mon% | 3.450±0.539 | 3.933±0.493 | 0.1362 |
| Gran% | 23.83±7.627 | 27.117±6.178 | 0.4249 |
| RBC | 5.830±1.829 | 4.668±1.349 | 0.2468 |
| HGB | 129.7±11.22 | 128±11.93 | 0.8082 |
| HCT | 39.73±2.200 | 40.62±1.623 | 0.4470 |
| MCV | 52.35±2.311 | 53.63±2.562 | 0.3837 |
| MCH | 18.92±2.025 | 19.50±1.517 | 0.5847 |
| MCHC | 342.5±23.28 | 349.8±18.68 | 0.5607 |
| RDW | 14.55±1.854 | 15.72±1.675 | 0.2794 |
| PLT | 816.7±217.3 | 840.2±216.9 | 0.8550 |
| MPV | 4.717±0.8519 | 4.517±0.5981 | 0.6480 |

WBC, white blood cell count, 10^9/L; Lymph, lymphocyte count, 10^9/L; MON, monocyte count, 10^9/L; Gran, granulocyte, 10^9/L; Lymph%, lymphocyte ratio, %; Mon%, monocyte ratio, %; Gran%, granulocyte ratio, %; RBC, white blood cell count, 10^12/L;HGB, hemoglobin, g/L; HGB, hemoglobin, g/L; HCT, hematocrit, %; MCV, mean corpuscular volume, fL; MCH, mean corpuscular hemoglobin, pg; MCHC, mean corpuscular hemoglobin concentration, g/L; RDW, red blood cell volume distribution width, %; PLT, platelet, 10^9/L; MPV, mean platelet volume, fL. Student's t test was applied to the statistical analysis. Data are mean ± SD, n=6.

Supplementary Fig. S1


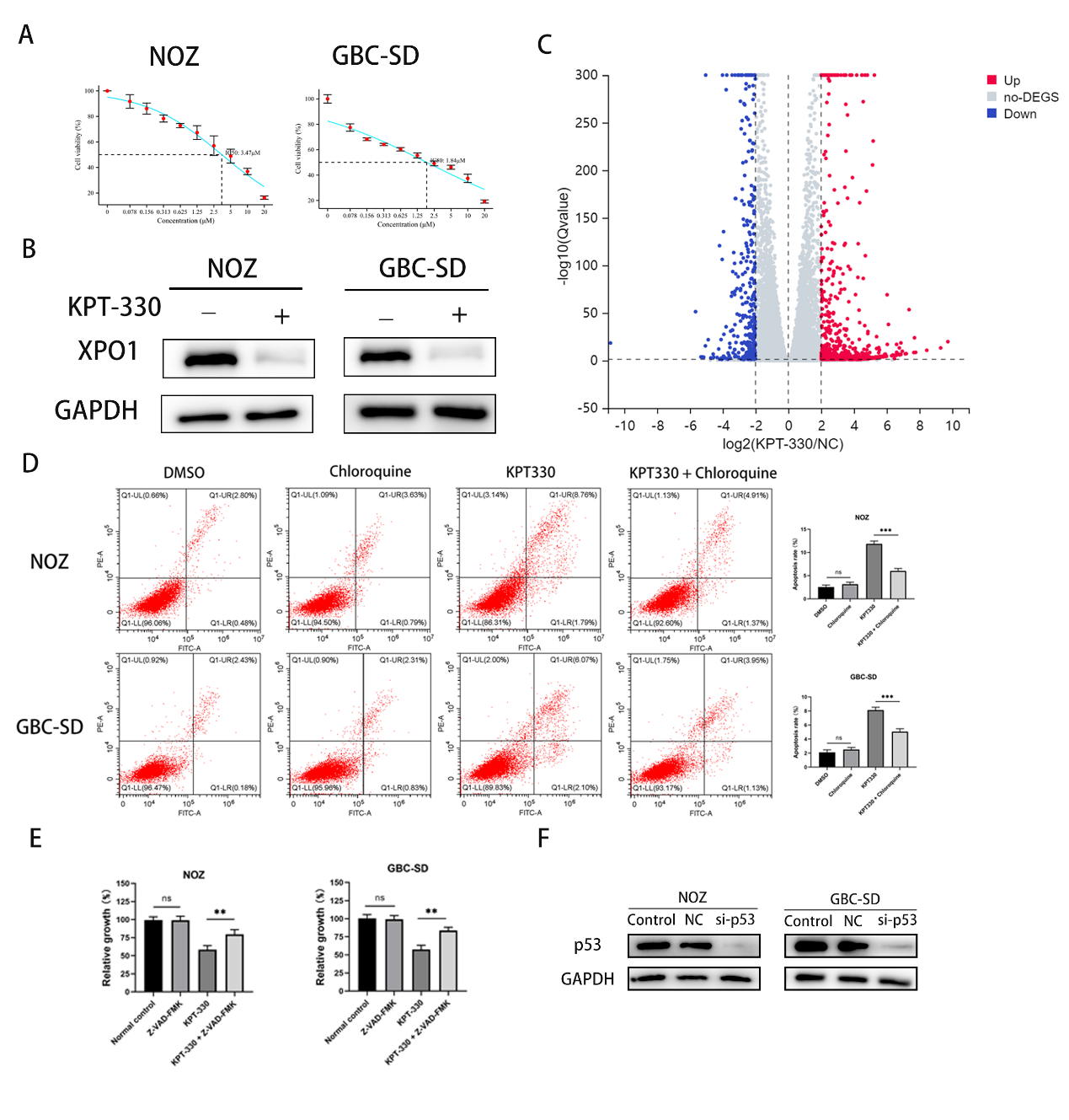


Supplementary Fig. S1

A. Cell sensitivity of NOZ and GBC-SD cells was evaluated by CCK-8 assays. Cells were exposed to KPT-330 for 48h and KPT-330 IC50 values for NOZ and GBC-SD cells were 3.47 and 1.84 μM, respectively. B. The XPO1 expression was detected by western blot after KPT-330 for 48h in NOZ and GBC-SD. C. Volcano plots of differentially expressed genes under the control of |${log}_{2}FC$| ≥ 2 and the Q-value ≤ 0.05 after KPT-330 treatment in NOZ cells. D. Flow cytometry using PI/Annexin V-FITC double stain of NOZ and GBC-SD after KPT-330 or chloroquine treatment. Cells were treated with chloroquine (concentration of 0.2 μM) for 6h, then were treated with KPT-330 for 48h. E. Z-VAD-FMK attenuated inhibition effects of KPT-330 in NOZ and GBC-SD assessed by cell proliferation assays. Cells were treated with Z-VAD-FMK (concentration of 0.1 μM) for 6h, then were treated with KPT-330 for 48h. F. The p53 expression after siRNA transfected NOZ and GBC-SD was detected by western blot. “NC” means “Negative Control” group transfected by NC-siRNA. “Control” means “untransformed cells” group. Student's t test was applied to the statistical analysis in this figure. Data presented as mean ± SD (n = 3).

Supplementary Fig. S2


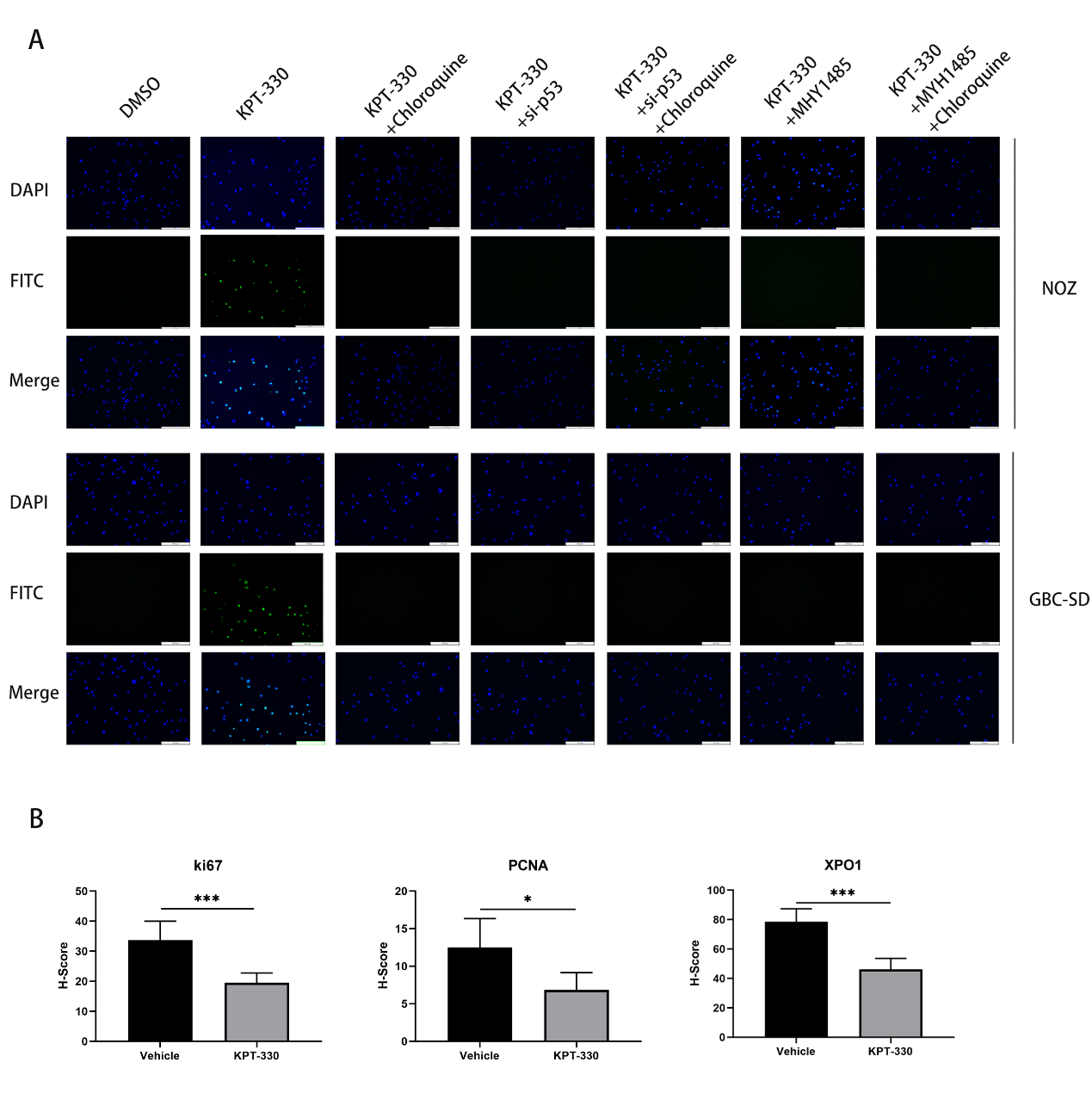


Supplementary Fig. S2

A. Fluorescence images of TUNEL after KPT-330 or p53-siRNA or MHY1485 or chloroquine treatment in NOZ and GBC-SD. NOZ and GBC-SD cells were pre-treated with p53-siRNA for 48h or MHY1485 (concentration of 0.5 μM) or chloroquine (concentration of 0.2 μM) for 6h, then were treated with or without KPT-330 for 48h. Scale bars represent 100 μm.

B. Immunohistochemistry score of XPO1, Ki67 and PCNA expression levels of xenograft tumor tissues in KPT-330 treatment or vehicle group. Student's t test was applied to the statistical analysis.
